# Evaluation and forecasting of siRNA delivery technologies: An analysis of hierarchical decision model based on patent data

**DOI:** 10.1016/j.omtn.2026.102943

**Published:** 2026-04-30

**Authors:** Shiyun Chen, Yixuan Peng, Dilei Yan, Liyang Lyu, Thomas Scherngell, Yuanjia Hu

**Affiliations:** 1 State Key Laboratory of Mechanism and Quality of Chinese Medicine, University of Macau, Macao SAR 999078, China; 2 Center for Innovation Systems & Policy, Austrian Institute of Technology, Vienna 1210, Austria; 3 Centre for Pharmaceutical Regulatory Sciences, Institute of Chinese Medical Sciences, University of Macau, Macao SAR 999078, China; 4 DPM, Faculty of Health Sciences, University of Macau, Macao SAR 999078, China

**Keywords:** MT: Delivery Strategies, siRNA, drug delivery technology, hierarchical decision model, patent evaluation, high-value patents

## Abstract

Small interfering RNA (siRNA) represents a transformative modality in next-generation therapeutics, yet challenges in delivery efficiency still limit its clinical translation. Systematic evaluation tools are needed to reveal technological progress in this area. Given the innovation momentum inherent in patents, this study develops a patent-based evaluation model for siRNA delivery technologies from a technology management perspective. By integrating the Hierarchical Decision Model (HDM) with patent landscape analysis, the model incorporates 15 multidimensional criteria across technological, commercial, and legal perspectives, with the commercial perspective assigned the greatest weight (41%), followed by technical (32%) and legal (27%) perspectives. A large-scale dataset comprising 20,319 siRNA delivery-related patent documents was quantitatively assessed using this model. The results reveal that high-value patents are primarily concentrated in lipid-based carriers and ligand-siRNA conjugates, with firms such as Alnylam and Arbutus emerging as dominant innovation leaders. Furthermore, the analysis highlights that therapeutic indication, assignee’s technological accumulation, technological impact, and drug delivery method are key drivers of patent value. Overall, the proposed model supports strategic decision-making in patent portfolio management and technology forecasting by identifying high-value innovations.

## Graphical abstract

**Figure:**
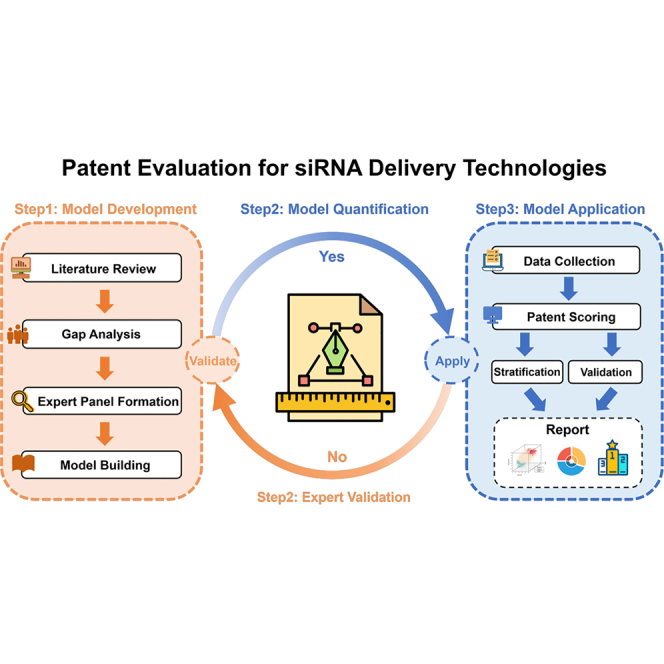

## Teaser

Small interfering RNA (siRNA) therapeutics depend heavily on efficient delivery technologies. This study develops a patent-based evaluation framework to identify high-value innovations in siRNA delivery. The results highlight key technological trends and leading innovators, providing insights to guide future development and strategic decision-making.

## Introduction

Despite decades of progress in molecular medicine, effective treatments for complex conditions like cancer and genetic disorders remain rare, mainly because traditional therapies cannot target so-called “undruggable” genes. Nucleic acid-based therapeutics, especially small interfering RNA (siRNA), have become a new paradigm, providing accurate, sequence-specific gene silencing.^1^ With six Food and Drug Administration-approved (FDA) siRNA-based therapies, the clinical value of siRNA has been partially validated. However, the effectiveness of *vivo* delivery remains the primary barrier to broader clinical applications. Systemic administration faces rapid degradation, immune clearance, poor cellular uptake, and inefficient endosomal escape.^2^^,^^3^ These technical challenges also increase the uncertainty of research and development (R&D) activities, exposing firms to high costs and a heightened risk of failure in decision-making.

In this context, proactively identifying technological trends and evaluating innovative potential are crucial for firms and research organizations. Patents serve as tangible indicators of technological innovation, providing valuable insights into the current state of technology development.^4^ By addressing issues such as market failure and knowledge spillovers, they provide legal support for innovation and bridge fundamental research and commercial applications.^5^ Through the temporary exclusivity granted by patent rights, they incentivize innovation and establish market-shaping barriers that influence competitive dynamics.^6^^,^^7^^,^^8^ Over the past decade, rapid advances in siRNA delivery have led to a sharp increase in related patents. However, high technological uncertainty and rapid innovation have created a significant gap between patent discovery and clinical translation. Therefore, developing systematic patent evaluation tools to enable the early identification of high-value patents has strategic significance to firms, institutions, and governments.

Given the complexity of siRNA delivery, multidimensional criteria facilitate the efficient identification of high-value patents. Multi-criteria decision-making (MCDM) methods have proven effective across engineering domains,^9^^,^^10^ although their application in the biopharmaceutical field remains limited. Meanwhile, big data-based patent landscape analysis effectively reveals overall patent structures within specific technological domains, as demonstrated in HER2-targeted therapies^11^ and induced pluripotent stem cells (iPSCs).^12^ While patent landscape analysis presents diverse indicators of macro-level trends, it often overlooks individual patents, limiting its usefulness in assessing patent-level value.

To address this gap, this study proposes an integrated evaluation tool that combines Hierarchical Decision Model (HDM) with patent landscape analysis to identify high-value patents in siRNA drug delivery from commercial, technical, and legal perspectives. We first conducted a patent landscape analysis to define evaluation perspectives and criteria. Domain experts then quantified their relative importance through pairwise comparisons, and desirability functions were used to assign quantitative scales for each indicator, enabling individual patent documents to be scored and ranked. This study aims to: (1) reveal promising siRNA delivery technologies, (2) support informed R&D investment decisions, and (3) promote sustained innovation.

## Results

### Variable input pool

The patent landscape analysis of siRNA delivery technologies provided multidimensional data support across various aspects, including temporal distribution, geographical coverage, citation relationships, and assignee characteristics. Based on the mode of data acquisition, the relevant variables could be categorized into two layers: the basic data layer and the network analysis layer.

The basic data layer included structured information directly extracted from patent databases and semantically annotated variables from manual indexing, providing stable, reproducible, and operable indicators for cross-study comparison and quantitative analysis. Representative metrics included patent family size, number of claims, International Patent Classification (IPC) codes, therapeutic indications, and drug delivery methods.

The network layer captured structural relationships among patents and assignees by using complex network modeling. In the patent citation network, degree and betweenness centrality quantified each patent document’s connectivity and intermediary role, highlighting core patents with significant influence. Meanwhile, the assignee collaboration network revealed interorganizational cooperation patterns, allowing assessment of each assignee’s technical involvement in siRNA delivery.

### Key perspectives influencing the evaluation

The study established an integrated evaluation framework for siRNA delivery patents, structured around three core dimensions: technological, legal, and commercial. Technological factors capture innovation potential, legal factors ensure exclusivity and protection, and commercial factors reflect market feasibility. Based on the patent landscape analysis, the expert panel selected 15 criteria—five for each dimension. The following sections describe these criteria and their roles in the evaluation.

### Technical perspectives

The evaluation of technical considerations for siRNA drug delivery patents mainly emphasizes the technology’s complexity, development stage, and influence. The early selection of drug delivery strategies has a decisive impact on whether an siRNA project can successfully advance to the clinical stage. The combined use of delivery technologies can simultaneously overcome multiple delivery challenges, thereby increasing the success rate of project translation from R&D to clinical applications. Patent citation is an important metric for measuring the dissemination and evolution of technology since it reveals the flow of technical knowledge. This study uses cumulative total citations instead of fixed-window citations to more robustly capture long-term knowledge diffusion and reduce instability for recently disclosed patents. A patent citation network is a network structure built on citation relationships.^13^ By performing a topological analysis of the network and using measures such as harmonic closeness centrality and clustering coefficient, it is possible to evaluate a patent’s technological maturity and position within its field. The translational evidence aims to assess whether the technology has entered a substantial translational stage by examining the *in vivo* and/or *in vitro* experimental data disclosed in the patent.

### Legal perspectives

At the legal level, patent value is primarily determined by the scope and strength of protection, reflected in various attributes. The range of IPC subclasses indicates the diversity of technical applications. The number of claims and the size of the family serve as key indicators of protection scope, with an appropriate claim count limiting competitors’ ability to bypass patents, and family size reflecting market coverage. Geographic coverage and patent age further capture the global extent and longevity of protection.

### Commercial perspectives

The commercial value of a patent reflects its market potential and ability to generate tangible benefits. In therapeutics, this is often linked to market size and trends; for example, cancer-related patents usually hold greater value than orphan drugs due to larger patient populations, urgent demand, and high treatment costs. Commercial value is also shaped by the assignee’s organizational type and capabilities: academic institutions drive basic research but rely on industry partners for commercialization, while experienced patentees with larger portfolios typically hold stronger market positions. Indicators further include collaboration networks and R&D experience in small nucleic acids; notably, companies with prior success in siRNA drug development demonstrate the expertise and capacity needed for commercialization.

**Figure 1:**
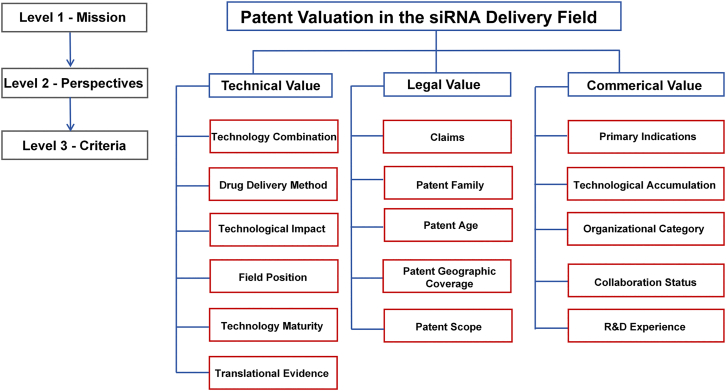
Patent indicator framework

All perspectives, criteria, and their definitions were used to construct the model for presentation to the expert panel for further validation and quantification. The model was fine-tuned to obtain the final version with the advice of the experts (Figure 1). Further,Table 1 provides a comprehensive overview of the definitions, measurement units, and reference sources for each criterion.

**Table 1:** Criteria of the perspectives affecting patent evaluation

Perspectives	Criteria	Definition	Reference
Technical	technology combination	this evaluates whether drug technologies are used in combination and, if so, how many combinations are present.	Hadinoto et al.,^14^ Peng et al.^15^
	drug delivery method	drug delivery methods can be classified, including ligand-siRNA conjugates, lipid-based carriers, chemical modifications, polymer carriers, biological carriers, etc.	Friedrich et al.^16^
	technological impact	the number of forward citations of a patent reflects its leading role in the industry.	Harhoff et al.,^17^ Narin et al.^18^
	field position	harmonic closeness centrality—a topological metric from the patent citation network—reflects a patent’s positional prominence by summing the reciprocals of its shortest distances to all other patents. Higher values denote greater centrality and influence within the field.	Esposito et al.^19^
	technology maturity	clustering coefficient—a topological metric from the patent citation network—measures the degree of interconnection among a patent’s neighboring patents. A higher value indicates a stronger internal knowledge linkage and a higher level of technological maturity.	Rendón de la Torre et al.,^20^ Yu et al.^21^
	translational evidence	*In vivo* and/or *in vitro* experimental data disclosed in a patent, demonstrating whether the technology has entered a substantial translational stage.	Seyhan et al.,^22^ Zhang et al.^23^
Legal	claims	number of patent claims	Pereira et al.^24^
	patent family	family patent count	Akinsolu et al.^25^
	patent age	time interval from patent filing date to the current date (in years)	Huang et al.^26^
	patent geographic coverage	the number of countries or regions with family patents	Kim et al.^27^
	patent scope	the number of IPC4 subclasses assigned to this patent	Fernández-Ribas et al.^28^
Commercial	primary indications	the main indications’ classification, such as cancer, inflammatory diseases, infectious diseases, and genetic and congenital disorders	Hu et al.,^1^ Friedrich et al.^16^
	technological accumulation	the number of patents owned by the assignee in the field	Chandra et al.^29^
	organizational category	the organizational category identifies the type of assignee associated with a patent, including entities such as companies, public institutions, universities, individuals, or joint entities.	Bhaskarabhatla et al.^30^
	collaboration status	the collaboration rate of the patent assignee in the field	Ma et al.^31^
	R&D experience	this assesses whether the patent assignee has a track record in which they have successfully developed small nucleic acid or siRNA-based therapies	Sung et al.^32^

### Weight distribution of perspectives and criteria in the HDM

The expert panel quantified HDM to evaluate the relative importance of 15 factors in identifying high-value patents. Inconsistencies and disagreements consistently stayed within the acceptable threshold of 10% throughout the process, requiring no adjustments (Table S1). At the first hierarchical level, the commercial perspective carried the most weight (41%), followed by technical (32%) and legal (27%) factors.

**Figure 2:**
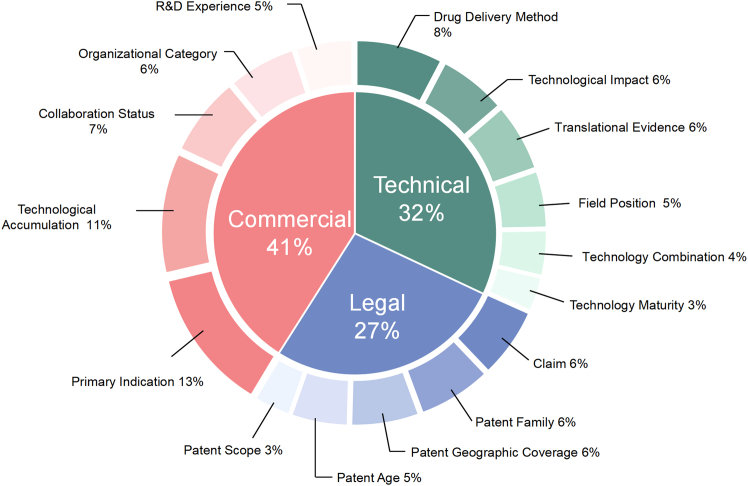
Pie chart of indicator weights

Among individual indicators, the primary indication of a drug was the most influential (13%), followed by the patent owner’s technological accumulation (11%) and drug delivery method (8%). Technological impact (forward citations) and translational evidence each contributed 6%. From the legal dimension, claim counts, family size, and geographical coverage were equally weighted. In contrast, the patent scope is minimal, with a weight of only 3%. Figure 2 presents the global weight distribution with the model. The ranked global weight distribution is further illustrated in Figure S2.

### Desirability curves of the criteria in the HDM

**Figure 3:**
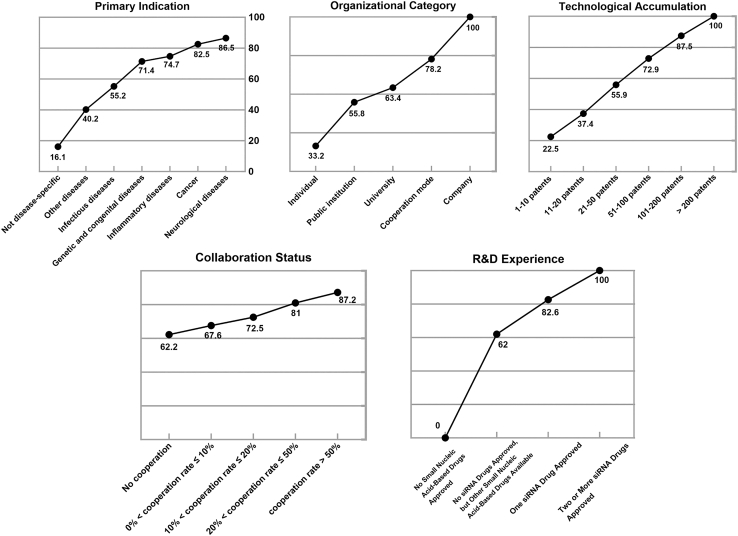
Desirability curves from the commercial perspective

From a commercial perspective, the most valuable patents exhibit the following characteristics (Figure 3): the primary indication targets neurological disorders, the assignee is a corporate entity, and the assignee holds numerous patents in the relevant field, ideally more than 200, and demonstrates a high level of technological collaboration. Moreover, an ideal licensee should possess a proven track record in drug development, particularly in the field of nucleic acid therapeutics such as siRNA, thereby demonstrating the professional expertise and technical capabilities required for successful drug development and commercialization.

**Figure 4:**
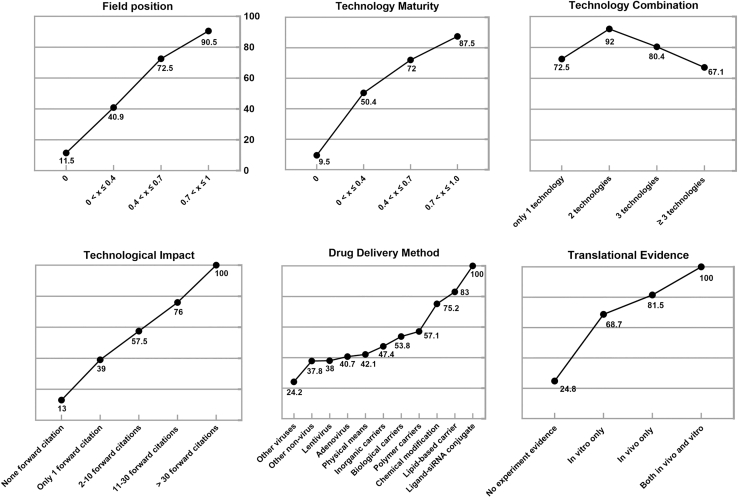
Desirability curves from the technical perspective

From the technical perspective, ligand-siRNA conjugates are regarded as the most ideal delivery methods for high-value patents. The patent that reports both *in vivo* and *in vitro* experimental data is considered to have the most convincing translational evidence. Furthermore, topological parameters—field position and adjacent technology—provide an intuitive reflection of a patent’s knowledge accumulation in innovation. A higher value indicates a stronger knowledge linkage between the patent and its technological domain. Interestingly, using three or more delivery technologies in combination is not considered a typical characteristic of high-value patents. This may be because complex formulation designs can significantly increase R&D costs and elevate the risk of side effects (Figure 4).

**Figure 5:**
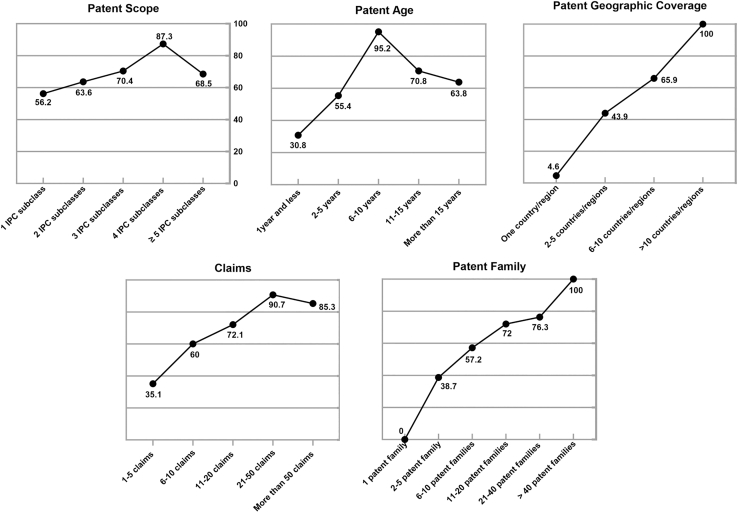
Desirability curves from the legal perspective

From a legal perspective (Figure 5), patents with broad geographical coverage and large family sizes are generally more competitive, as wider protection improves global enforceability and commercial potential. However, having more claims does not always add value; the optimal range for high-value patents is 21–50, balancing core technology protection with manageable legal complexity. Similarly, 6–10 years patents are most advantageous, as they reflect technological maturity while offering a significant protection period.^33^ An IPC count of four also appears optimal, as excessive classifications may dilute technological focus and complicate market positioning.

### Patent value scoring for siRNA delivery

Following validation and quantification, the model was applied to a real-world case study to demonstrate its practical utility. A total of 20,318 siRNA delivery patent documents were evaluated using a 15-indicator model, in which weighted sums were used to generate a “desirability scoreˮ for each patent.

**Figure 6:**
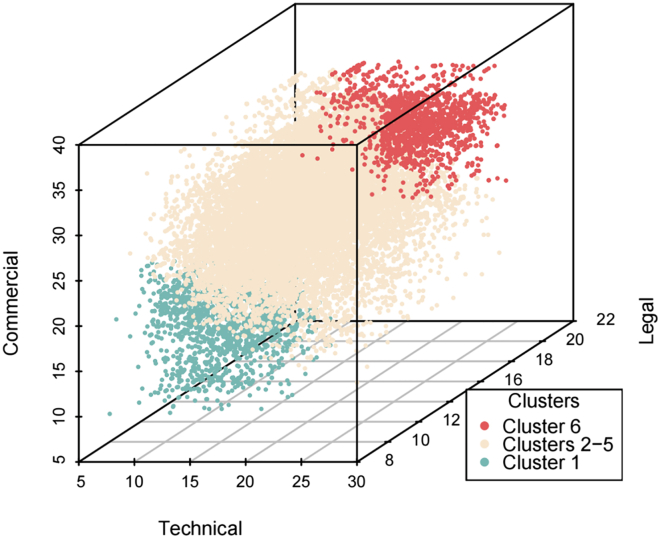
Scatterplot of score distribution

A 3D scatterplot (Figure 6) was created to illustrate the score distribution across the different dimensions. The *x-*, *y-*, and *z*-axes represent weighted scores in the technical, commercial, and legal dimensions, respectively. The results show that, in the commercial dimension, the highest score is 37.977 and the lowest is 10.914, with an average of 26.078. Legal scores range from 5.608 (lowest) to 24.821 (highest), averaging 16.992. Technical scores span from a low of 7.964 to a high of 28.607, with a mean of 14.193. These distributions reflect significant variability in performance and demonstrate diversity and competitiveness in the siRNA delivery patent landscape. According to the model, a “perfect” patent would achieve a theoretical maximum score of 95 across all 16 indicators. In practice, however, the highest observed score is 82.511, which is approximately 13.15% below the maximum. This indicates that no “perfectˮ patent has yet been identified under the current evaluation criteria.

To further analyze the characteristics of patent overall scores, this study applied the natural breaks classification method, which detects natural groupings in continuous data by minimizing intragroup variance and maximizing intergroup differences. This method produced six clusters: cluster 6 contains the top-scoring patents, while cluster 1 includes those with the lowest scores. In addition, clusters 2 through 5 represent intermediate performance levels. For ease of visualization, color coding was used: cluster 6 in red, cluster 1 in blue, and clusters 2–5 in yellow. Table S2 presents the mean and standard deviation of the overall scores, along with the technical, commercial, and legal scores for each cluster.

### High-value patent characteristics

**Figure 7:**
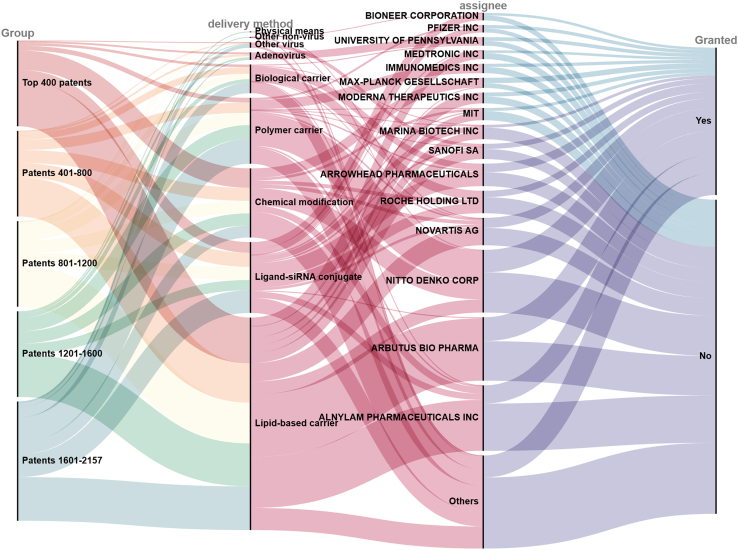
Sankey diagram of the top-ranked patent cluster (cluster 6)

This section presents an analysis of the characteristics of high-value patents in the field of siRNA drug delivery. This study focused on the top-ranked cluster 6, which comprised 2,157 patent documents. These patents were categorized into five distinct groups based on their value rankings. Sankey diagrams were used to visually represent the distribution of key features, including groupings, drug delivery methods, patent assignees, and patent grant status (Figure 7).

In the highest-ranked cluster 6, lipid-based carriers emerged as the dominant delivery method, accounting for the highest proportion with 993 patent documents. This prominence was attributed to their superior encapsulation efficiency and exceptional performance in both *in vitro* and *in vivo* expression. The successful commercialization of Onpattro from Alnylam Pharmaceuticals (the first FDA-approved siRNA therapeutic) and coronavirus disease 2019 (COVID-19) mRNA vaccines^34^ has further underscored the significance of lipid-based carriers as a core platform technology for nucleic acid drug delivery. Moreover, the continuous development of novel cationic lipids and targeted lipids has reinforced their critical role in advancing drug delivery systems. Ligand-siRNA complexes (331 patent documents) follow closely, with N-acetylgalactosamine-conjugated (GalNAc-conjugated) delivery systems emerging as the dominant strategy for small nucleic acid drug delivery. This system achieves highly specific liver targeting by binding to asialoglycoprotein receptors (ASGPRs) on hepatocytes.^35^ This technology has facilitated the development and approval of several siRNA-based drugs, including Givosiran, Lumasiran, Inclisiran, and Vutrisiran. Chemical modifications, with 323 patents, rank third in terms of delivery methods. These modifications are categorized into three main types: phosphate backbone, ribose, and base modifications. Chemical modifications are crucial for improving the stability, specificity, and overall therapeutic efficacy of siRNA, addressing inherent limitations, such as poor stability and safety concerns. Alternative delivery methods, including biological carriers and physical approaches, account for a smaller fraction of delivery strategies but provide versatile functionalization options to enhance siRNA release. These methods also potentially broaden the scope of extrahepatic delivery applications.^36^ Polymer-based delivery systems (308 patent documents) also represent a significant portion of siRNA delivery strategies. Polymer carriers offer an effective approach to enhancing the cellular uptake of siRNA. For instance, cationic polymers can interact with negatively charged siRNA via electrostatic interactions, effectively protecting siRNA from enzymatic degradation.^37^

As extrahepatic delivery and tumor targeting have become the most active areas in recent siRNA delivery research,^38^ we further analyzed the number of relevant patents in cluster 6. The results show that cluster 6 contains 208 extrahepatic delivery patents (compared to 111 trahepatic delivery patents) and 1,178 patents related to tumor diseases. Based on these findings, we conducted a stratified analysis and constructed delivery route-time and disease category-time trajectory models (Figure S3). The results demonstrate that the use of extrahepatic delivery and tumor-targeted siRNA strategies is steadily increasing, reflecting growing interest in expanding the use of siRNA therapies beyond liver-specific applications. Expanding treatment indications to include tumors is expected to significantly enhance the market potential of these advanced delivery platforms.

Furthermore, this study analyzed the distribution of high-value patent ownership in the siRNA drug delivery field. The results reveal that among the top 16 assignees, 13 are multinational pharmaceutical companies or specialized biotechnology firms, while the remaining three are represented by the Massachusetts Institute of Technology (MIT), Max Planck Society, and the University of Massachusetts. This distribution indicates that industrial entities dominate the innovation system for siRNA delivery technologies, whereas academic institutions and public agencies are relatively underrepresented.

Notably, Alnylam Pharmaceuticals and Arbutus Biopharma have emerged as leading players. Among them, Alnylam holds the largest share with 306 patents. It is followed by Arbutus with 297 patents. On the one hand, as a leader in RNA interference (RNAi) therapeutics, Alnylam currently has five approved and marketed siRNA products. The company excels in mainstream siRNA delivery technologies, supported by a well-established patent portfolio. Arbutus, on the other hand, focuses on the development of combination therapies for chronic hepatitis B. Additionally, Arbutus has built a significant and diverse portfolio of core patents in the lipid nanoparticle (LNP) technology space, encompassing a wide range of patents related to cationic lipids and polyethylene glycol (PEG) lipids. These patents cover compound structures, formulations, LNP compositions, and various applications. Moreover, patent authorization data reinforce the analysis of high-value patent characteristics. These data confirm that granted patents are predominantly concentrated in two delivery technologies—lipid-based carriers and ligand–siRNA conjugates—with assignees significantly clustered in Alnylam and Arbutus.

For clarity, Table S3 lists the top 50 ranked patent families, including numbers, assignees, technology summaries, and drug delivery methods. Building on this dataset, the “discussion” section further analyzes several representative high-scoring patents through detailed case studies.

### Sensitivity analysis

Sensitivity analysis is a method used to assess the impact of each input parameter on the results and to evaluate the rationality of the selected indicator system.^39^^,^^40^ The higher the sensitivity, the more unstable the evaluation results will be. In this study, sensitivity analysis is applied to investigate the impact of each input parameter on the patent scoring.

(Equation 1) $$S_{i}=\frac{\left|\left(V_{i}/N\right)-\left(v_{i}/n\right)\right|}{V_{i}}\times 100\%\text{.}$$

*S*_*i*_ represents the sensitivity of *i*th evaluation unit, *V*_*i*_ represents the patent score of *i*th evaluation unit, *v*_*i*_ represents the patent score after removing *i*th input index, “*N*” and “*n*” are the number of parameters used to calculate *V*_*i*_ and *v*_*i*_, respectively.

The impact of removing each input parameter on the patent evaluation model (Figure S4) indicates that the average sensitivity of all parameters is generally low, ranging from 0.10% to 0.64%. Removing primary indication has the most significant impact on the model, followed by technological accumulation, with an average sensitivity of 0.45%. In contrast, the change in patent age has the smallest impact, with an average sensitivity of 0.049%. The influence of removing parameters on the patent score correlates well with the weight of each indicator. For instance, the combined weights of primary indication, technological accumulation, and R&D experience are 0.13, 0.11, and 0.05, respectively. The results of the parameter sensitivity analysis indicate that the model is relatively stable, as removing any single criterion does not significantly impact the siRNA delivery patent value assessment. This suggests that the model does not overly depend on any single parameter, with all input criteria contributing to the overall evaluation.

In addition to parameter-level sensitivity analysis, a ranking-level analysis was conducted to assess the robustness of patent rankings across different weighting schemes. Specifically, ±10% random perturbations were applied to indicator weights, followed by 1,000 Monte Carlo simulations. The consistency between the original ranking and the perturbed rankings was extremely high, with a mean Spearman’s correlation coefficient of 0.99925 (SD = 0.00035; minimum = 0.99761), indicating negligible changes in the overall ranking structure. Furthermore, the stability of high-value patents was evaluated using the top patents identified in the original model. The mean top—2,157 stability probability was 0.98044 (SD = 0.00521; range = 0.96477–0.99351), indicating that most high-value patents remained consistently within the top across weight perturbations. These results are further supported by the histograms presented in Figure S5, which demonstrate the stability of the rankings under different perturbation scenarios. These results collectively confirm that the evaluation model is highly robust to reasonable variations in weighting schemes, with stable and reliable identification of high-value patents.

## Discussion

### Promising siRNA delivery technologies

The development of delivery technologies is primarily motivated by the need to overcome several ongoing barriers. The challenges include accumulation in the target tissue or organ,^41^ effective cellular uptake,^42^ and endosomal escape.^43^ Although various novel delivery strategies have been developed,^44^ only lipid-based carrier systems and ligand-based conjugate technologies have achieved commercial success. This study evaluated siRNA delivery technologies from a patent perspective, and this section offers a detailed discussion of key delivery methods, along with selected high-score patents that showcase important technological progress.

Patisiran, the first siRNA drug approved for clinical use, demonstrated the effectiveness of LNP platforms.^45^ As the most well-established delivery system to date, LNPs have shown significant benefits in enhancing siRNA stability and improving *in vivo* delivery. However, some limitations remain. For instance, traditional LNPs composed of permanently charged cationic lipids tend to interact nonspecifically with plasma proteins and have relatively high immunogenicity, which can reduce therapeutic effectiveness^46^ and increase systemic toxicity.^47^ To overcome these limitations, many pharmaceutical companies and research organizations have recently focused on developing new ionizable lipids^48^ and hybrid LNPs^49^ to improve the efficiency and targeting of siRNA delivery. Hybrid LNPs have demonstrated superior gene-silencing efficiency and decreased systemic toxicity, especially in cancer treatments.^50^ Meanwhile, ionizable lipids show improved endosomal escape abilities due to their structural flexibility and ease of manufacturing scalability.^51^ The results of this study’s patent value assessment system further support these technological advances. Highly ranked patents in this system include the patent US20050175682A1 from Arbutus Biopharma (score: 77.067), which marks a breakthrough in hybrid LNP delivery technologies. Sanofi’s patent US20170240501A1 (score: 77.648); and Alnylam’s patent WO2010144740A1 (score: 81.498), both of which demonstrate progress in novel cationic lipid technologies.

Ligand-conjugated delivery systems using GalNAc are another major breakthrough, with five of the six FDA-approved siRNA therapeutics employing this technology. This technology allows targeted delivery to hepatocytes by specifically recognizing the ASGPR, which is highly expressed on liver cell surfaces but minimally present in other tissues.^52^ Through ASGPR-mediated endocytosis, GalNAc-conjugated siRNAs achieve efficient uptake into hepatocytes, providing greater tissue specificity and reduced immunogenicity compared to traditional lipid-based delivery systems. As a result, GalNAc-siRNA conjugation has become the leading strategy for liver-targeted RNAi therapeutics. However, delivering siRNA beyond the liver remains difficult because of poor endosomal escape after cellular uptake, which restricts its wider therapeutic use.

However, the technological trajectory of siRNA therapeutics is not static. The results of the layered analysis, conducted by constructing delivery route-time and disease category-time trajectory models, reflect a growing effort to expand siRNA-based therapeutics beyond liver-specific applications. Current research is actively exploring new delivery methods, such as new ligands covalently linked to siRNA.^53^ These ligands consist of small molecules, fatty acids, peptides, aptamers, antibodies, and saccharides.^41^ For example, the development of a C16 platform that uses short lipid chains attached to siRNA for targeted delivery to tissues such as the central nervous system, lungs, and eyes shows promise.^54^ This platform is currently undergoing clinical trials for Alzheimer’s disease and cerebral amyloid angiopathy. Another promising approach for extrahepatic delivery is the use of antibody-oligonucleotide conjugates (AOCs), which combine the targeting ability of antibodies with the therapeutic potential of oligonucleotides.^55^^,^^56^ AOCs enable precise delivery of gene-regulating drugs to specific tissues, similar to antibody-drug conjugates (ADCs) but with a focus on genetic modulation rather than cytotoxic payloads. Several AOC-based therapies are currently advancing into phase 3 clinical trials for the treatment of muscle diseases.

These ongoing innovations in extrahepatic are directly reflected in several high-value ligand-siRNA conjugate patents. For instance, patent US20160030585A1 (score: 76.965) discloses a fatty acid-siRNA conjugation system that enhances the pharmacokinetic properties and tissue-targeting capabilities of siRNA. Another example is patent US8709483B2 (score: 74.517), which describes a delivery strategy using an antibody targeting prostate-specific membrane antigen (PSMA) to enable the targeted delivery of siRNA to prostate cancer cells. These innovations, through diverse ligand designs, promise to expand the therapeutic potential of RNAi beyond liver-related diseases.

Polymer-based delivery systems rank third in prominence among high-value patents. Utilizing the proton sponge effect to help siRNA escape from endosomes, polymers offer a key strategy for overcoming endosomal escape challenges.^57^ However, the common cytotoxicity of these materials has led to the development of new low-toxicity polymers for safer siRNA delivery.^58^ Notably, Arrowhead’s patent US8658211B2 (score: 78.003) describes a stimuli-responsive, reversibly masked polyconjugate that improves the safety, stability, and effectiveness of siRNA delivery. Beyond formulation strategies, chemical modifications that change the molecular structure of siRNA are commonly used to improve *in vivo* stability and delivery. These modifications usually target the phosphate backbone, ribose sugar, or nucleobase. Phosphorothioate (PS) linkages are frequently used to boost nuclease resistance and extend half-life,^59^ while sugar modifications, such as 2′-O-methyl (2′-OMe) and 2′-fluoro (2′-F), enhance thermal stability, and reduce innate immune activation.^60^ The analysis of high-value patents shows that current siRNA candidates often combine multiple site-specific modifications to enhance stability and target accuracy.

Collectively, siRNA delivery technologies are evolving toward increased efficiency, improved tissue targeting, and lower toxicity. Approaches such as lipid-based carriers, GalNAc conjugation, polymer-based carriers, and chemical modifications offer unique advantages and have shown considerable promise in approved therapies and valuable patents. Looking ahead, extrahepatic delivery strategies are expected to advance through various approaches, aiming to expand the application of RNAi therapies beyond liver-related diseases and thereby benefit a broader patient population.

### From patents to approved drug: Translation and challenges

This study not only identifies high-value patents based on quantitative indicators but also examines their translational outcomes. Specifically, we analyzed which patents were directly associated with clinically approved siRNA drugs and explored why other high-value patents have not yet led to drug approval.

In this study, we consulted the FDA Orange Book website and extracted relevant patent data for FDA-approved siRNA drugs.^61^ We found that patents directly related to drug approval frequently appeared in the highest-ranked cluster 6. For example, US9394234B2, covering LNP delivery systems, is part of the patent portfolio supporting Onpattro, the first FDA-approved siRNA drug. This patent represents a core delivery technology that enabled stable systemic siRNA administration, thereby playing a decisive role in regulatory approval. Similarly, US8372968B2 (nucleic acid chemical modification) and CN101616677 B (liposomal formulation) are composition and formulation patents associated with Onpattro and were also ranked as high-value patents in cluster 6. Similarly, US9943539B1, a core patent for Givlaari, involves chemical modifications of siRNA, particularly methoxy modifications that enhance its stability. This technology was pivotal in the development of Givlaari, which was approved by the FDA in 2019 for the treatment of acute hepatic porphyria (AHP). US9814777B2, the core patent for the siRNA drug Amvuttra approved by the FDA in 2025, also appears in cluster 6. Amvuttra utilizes GalNAc ligands to deliver siRNA directly to liver cells for the treatment of cardiomyopathy of transthyretin-mediated amyloidosis (ATTR-CM). These high-value patents together underscore the importance of innovation and patent protection in the clinical translation of siRNA therapies.

However, not all high-value patents have led to clinically approved drugs. Several factors may explain this divergence. First, many patents cover early-stage platform technologies that are still in preclinical or early clinical development. Technical challenges such as stability, delivery efficiency, and scalable manufacturing may delay or prevent clinical translation.^41^ Beyond these intrinsic technical limitations, novel delivery systems must also compete with well-established platforms, such as lipid nanoparticles (LNPs). They therefore must demonstrate clear advantages in targeting efficiency, immunogenicity, or cost-effectiveness to justify further clinical validation. Significantly, the successful commercialization of siRNA drugs depends not only on innovations in delivery technology but also on factors such as sequence design^52^ and target selection.^62^ The design of specific siRNA sequences directly influences the drug’s targeting specificity and silencing efficiency. Furthermore, the commercialization of siRNA is also influenced by legal and patent strategies. While some delivery technologies are considered high-value patents, a drug’s patent portfolio often encompasses innovations across multiple domains. Due to patent barriers and cross-sector patent competition, certain technologies may face challenges with patent licensing and cross-licensing, further delaying drug approval.^63^^,^^64^

### Model validation and interpretation

This study conducted empirical validation using three external benchmarks: (1) patent litigation data, (2) a set of recognized “milestone” patents, and (3) patents related to currently marketed products. These analyses assessed the model’s capacity to identify patents with commercial potential and technological impact.

The litigation status of 20,319 patents was first examined, drawing on the widely supported premise that patent litigation is not random and is closely correlated with patent value.^65^ Due to their significant market relevance and economic potential, high-value patents are more likely to become subjects of legal disputes.^66^ Therefore, if this study’s model effectively identifies valuable patents, it should assign higher scores to those involved in litigation. According to records from the Derwent patent database, 16 patents in this study’s dataset have been involved in litigation in the US. Further analysis revealed that 13 of these 16 litigated patents ranked within the top 5% of the model’s output. Notably, several of these patents, including US8492359B2, US9404127B2, US9364435B2, and US9504651B2, originated from Arbutus Biopharma and serve as key parts of its LNP platform. These patents include lipid formulation optimization, component ratio design, and scalable manufacturing processes, which are areas that are central to technological competition in this field.

Additionally, this study incorporated a benchmark list of milestone patents identified by domain experts. All 10 milestone patents in siRNA drug delivery mentioned in Chen’s^67^ research (e.g., WO2002044321A2, WO2005007196A2, and WO2010042877A1) ranked in the top 10% of patents identified by this study’s model. These patents cover key technical domains, including lipid carriers (7 patents), chemical modifications (2 patents), and siRNA conjugates (1 patent). The strong alignment with recognized breakthroughs supports the model’s ability to capture patents’ commercial and technological significance.

To further validate the model’s performance, we selected patents related to FDA-approved siRNA drugs as a validation set, consulting the FDA Orange Book. This resulted in 291 delivery-related patent documents, chosen for their reflection of real-world technological, economic, and legal value and for having successfully navigated the commercialization process. These patents provide a robust benchmark for assessing the model’s ability to identify high-market-value patents. The analysis showed that marketed patents were significantly enriched in the higher ranking intervals, with a mean score of 70.99 and a median of 71.76 (Figure S6). A Mann-Whitney *U* test revealed a significant difference between marketed patents (mean = 70.99) and other patents (mean = 57.06), with a *p* value of <0.00001, further demonstrating the model’s capacity to distinguish high-value patents. Moreover, a chi-square test of patent classification into value tiers showed a significant enrichment of marketed patents in the high-value tier, supporting the model’s ability to differentiate patent value (Table S5).

### Practical implications

The evaluation tool developed in this study provides both methodological and practical benefits. Methodologically, it integrates three core dimensions-technical potential (e.g., delivery techniques, technology integration, and technological influence), legal robustness (e.g., patent family size and claim number), and commercial viability (e.g., indications and R&D experience), to form a balanced patent evaluation framework.

Practically, the tool benefits multiple stakeholders. For pharmaceutical companies and research institutions, it supports R&D resource allocation, technology transfer planning, and competitive benchmarking. For investors and policymakers, it provides a standardized, data-driven assessment framework that facilitates targeted resource allocation. This is particularly valuable in the biopharmaceutical industry, where substantial investment and information asymmetry are common challenges.

### Limitation

It must be acknowledged that patent evaluation has several limitations. First, they provide an indirect measure of efficacy, as differences in disease indications, treatment goals, and evaluation methods across patents complicate direct comparisons. Efforts to standardize these metrics often fail to fully address these challenges. Second, patent data are inherently retrospective, with a time lag between filing and publication. As a result, patent analyses tend to reflect past technological developments and may not capture ongoing research or emerging trends accurately. Finally, due to their indirect and retrospective nature, patent metrics have limited predictive power for future biological or clinical success. While they offer valuable insights into past innovations, they cannot replace the need for experimental validation or prospective studies to assess clinical outcomes. These limitations must be considered when interpreting patent data in biological and clinical research.

### Conclusion

By integrating technical, legal, and commercial dimensions through the HDM approach, this study establishes a systematic framework for assessing patent value. Applying this model to over 20,000 siRNA-related patents enables a data-driven identification of high-value innovations in siRNA drug delivery. The findings highlight key technological groups, such as lipid-based carriers and siRNA conjugates, along with concentrated patterns of patent ownership, offering insights into the competitive landscape and innovation trends within this emerging field.

## Materials and methods

### Variables

Patent landscape analysis serves as a tool for variable selection by systematically extracting and analyzing patent data within a specific technological field. The process follows a structured workflow that combines quantitative indicators with qualitative dimensions. This methodology provides multidimensional insights into patents and assignees, including:

(1) Temporal and spatial characteristics of patents.
(2) Patent technology categories and scope of legal protection.
(3) Patent citation relationships and network structure.
(4) Assignee profiles and collaborative network structure.

From a data perspective, the results can be divided into two dimensions: a basic data layer and a network analysis layer. The basic data layer covers structured variables such as technical fields, IPCs, and claim counts, which can be directly extracted from patent databases or manually annotated. In contrast, the network analysis layer involves constructing citation and collaboration networks for deeper exploration. This study incorporated patent landscape analysis results into HDM as input variables.

### HDM

The model was developed by an expert panel responsible for structural validation and quantification. The panel assessed the relevance of hierarchical perspectives to the problem objectives and performed pairwise comparisons to assign weighted priorities to criteria. These weights were combined across the hierarchy to derive optimal solutions. Each patent was then evaluated based on these criteria, resulting in a final score (*P*) ranging from 0 to 100. In this study, the patent score *P* was determined by the following:

(Equation 2) $$P=\sum_{k=1}^{K}\sum_{jk=1}^{JK}P_{k}\times C_{jk}\times D_{jk}\text{,}$$

where *P* represents the patent score, *K* represents the number of perspectives, *J* signifies the number of criteria, *P*_*k*_ signifies the weight of the *k*-th perspective, *C*_*jk*_ represents the importance of the *j*-th criterion under the *k*-th perspective, and *D*_*jk*_ signifies its desirability value of criterion for perspective. The overall patent score *P* was calculated by integrating the weights and desirability values of the perspectives and their criteria. The specific model implementation steps are described below.

First, the expert panel was formed by reviewing authoritative sources (PubMed and Google Scholar) and peer recommendations, using snowball sampling to expand the pool. Experts were selected for their expertise in siRNA, drug delivery, or IP, based on professional background, academic contributions, conflict-free status, and willingness to participate. In total, 23 experts were involved: 15 in validation and 20 in quantification, representing academia, industry, and government (Table S4). Then, the experts identified the most relevant perspectives and criteria, which were incorporated into the HDM model.

Next, experts completed pairwise comparison questionnaires (supplemental questionnaire S1) to assess the relative importance of perspectives and criteria. Their inputs were aggregated into local weight coefficients (within-level importance) and global priority indices (overall contribution), expressed as percentages.

Patent evaluation and ranking employed a desirability function. Each criterion was scored on a 0–100 scale, validated through expert questionnaires (supplemental questionnaire S2) to construct desirability curves. Patent attributes were mapped to these curves to calculate component scores; for multi-tiered criteria, average desirability was used. Finally, component scores were weighted by global priorities and summed linearly to generate overall patent value scores.

Notably, experts evaluating the HDM occasionally provided judgments that contradicted their earlier assessments, mainly due to subjective bias or differences in understanding during quantification. To ensure reliability, the HDM imposed a consistency threshold of 10%; experts exceeding this limit were asked to review and revise their judgments.

Beyond individual consistency, the level of disagreement among experts was also assessed. Although some divergence is expected, the model required a minimum level of consensus. Therefore, a 10% disagreement threshold was applied to identify group-level variability that could affect model stability and prompt further coordination.

### Data

This research utilizes the Derwent Innovation Platform for the integration of patent literature (https://clarivate.com/products/derwent-innovation/). The patent search terms were formulated as follows:

CTB = (siRNA∗ OR (small ADJ interfer∗ ADJ RNA∗) OR (short ADJ interfer∗ ADJ RNA∗) OR (silenc∗ ADJ RNA∗)) AND CTB = (deliver∗ OR vehicle OR carrier) AND PY ≥ (2002) AND PY ≤ (2022).

The patent selection process is illustrated in the flowchart (Figure S1), which outlines the number of patent documents at each stage, along with the exclusion criteria applied. In this study, individual patent documents were used as the fundamental unit of analysis. Following this selection procedure, a total of 20,319 patent documents were included in the final dataset.

### Framework

The overall research design was structured into three main components: model development and validation, model quantification and analysis, and model application. In the first phase, a literature review identified research questions and gaps, and candidate variables were derived from the patent landscape. An expert panel then validated the proposed perspectives and criteria using questionnaires. The second phase involved quantitative analysis, where experts performed pairwise comparisons at both perspective and criterion levels, and assigned desirability scores (0–100). In the third phase, the validated model was applied to patent documents related to siRNA drug delivery.

## Data and code availability

The raw data supporting the conclusions of this article will be made available upon request to the corresponding authors.

## Acknowledgments

The authors would like to thank Jialing Zhong and Yicong Ding from the University of Macau for their assistance with data cleaning.

This research was supported by the Science and Technology Development Fund of Macau SAR (no.: 0002/2025/NRP, 0008/2025/EQP, and 0049/2024/AGJ), and the 10.13039/501100004733University of Macau (no.: MYRG-CRG2023-00007-ICMS-IAS, MYRG-GRG2024-00268-ICMS-UMDF, UMDF-TISF/2025/011/ICMS, and SHMDF-AI/2026/003 with Dr. Stanley Ho Medical Development Foundation).

## Author contributions

S.C. performed validation, investigation, formal analysis, and visualization, and drafted the manuscript; Y.P. curated the data, developed the software, and contributed to manuscript writing and visualization; D.Y. contributed to data curation and investigation; L.L. contributed to methodology development and manuscript revision; T.S. provided supervision and contributed to writing and editing; Y.H. conceived the study, supervised the project, and contributed to writing and editing.

## Declaration of interests

The authors declare no competing interests.

## Supplemental information

Document S1. Figures S1–S5 and Tables S1, S2, S4, and S5

Data S2. Questionnaires

Table S3 Component score distribution. Detailed information on the top 50 ranked patent families

Document S3. Article plus supplemental information
