# Supplementary material for: Evaluation and forecasting of siRNA delivery technologies: An analysis of hierarchical decision model based on patent data
Source: Mol Ther Nucleic Acids. 2026 Apr 30;37(2):102943. doi: 10.1016/j.omtn.2026.102943 (PMC13213759; doi:10.1016/j.omtn.2026.102943)
Supplement: Document S1. Figures S1–S5 and Tables S1, S2, S4, and S5 [file mmc1.pdf]

OMTN, Volume *37*

Supplemental information

Evaluation and forecasting of siRNA delivery technologies: An analysis of hierarchical decision model based on patent data

Shiyun Chen, Yixuan Peng, Dilei Yan, Liyang Lyu, Thomas Scherngell, and Yuanjia Hu

Supplemental Questionnaires

Questionnaire 1 and Questionnaire 2 are provided as a separate supplemental file under the "Supplemental Videos and Spreadsheets" category.

Supplemental Figures

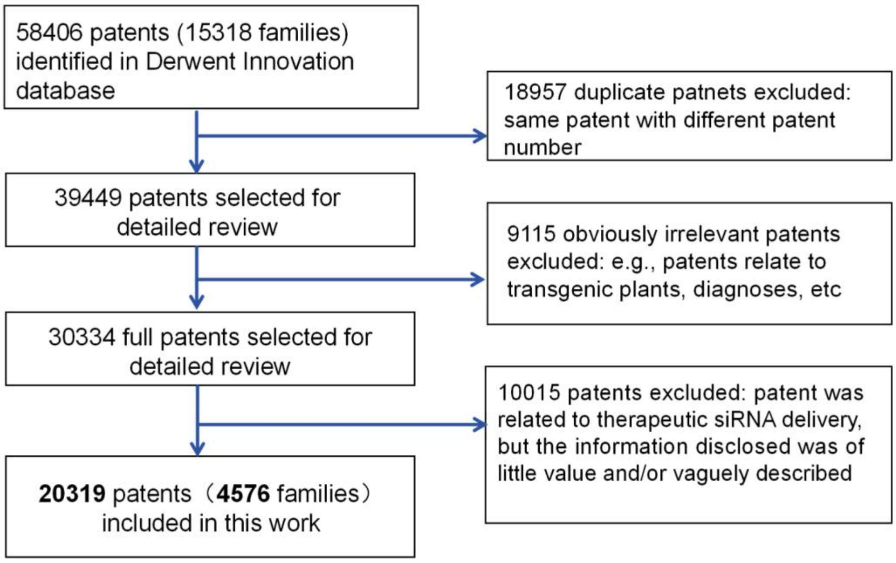

Figure S1. Patent screening process flowchart

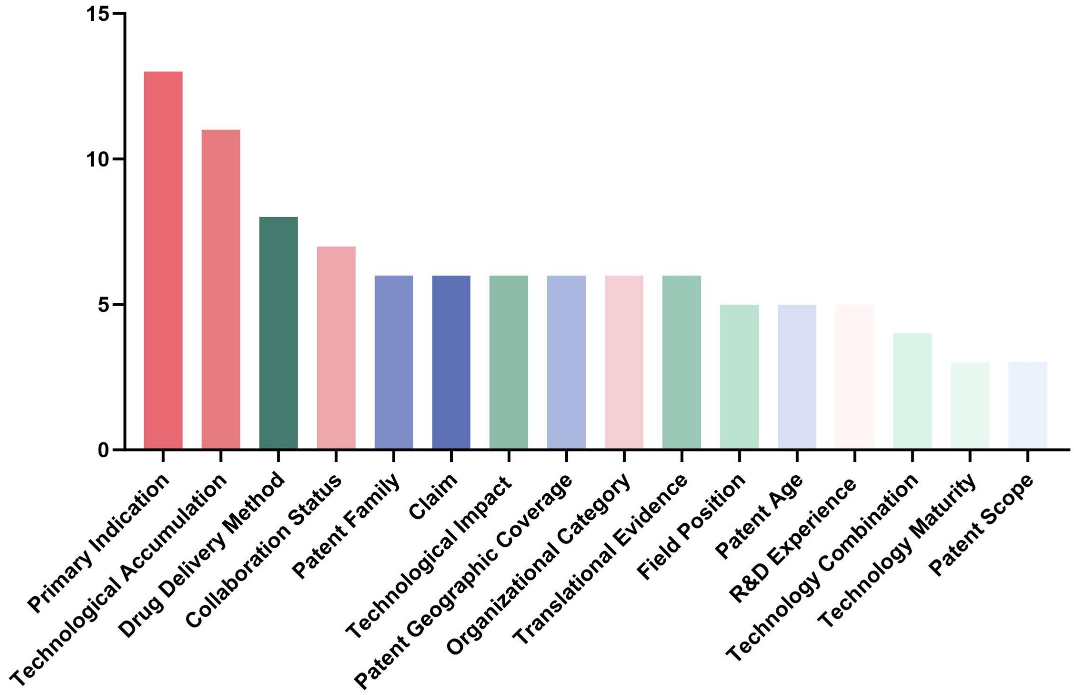

Figure S2. Sorted global weight distribution

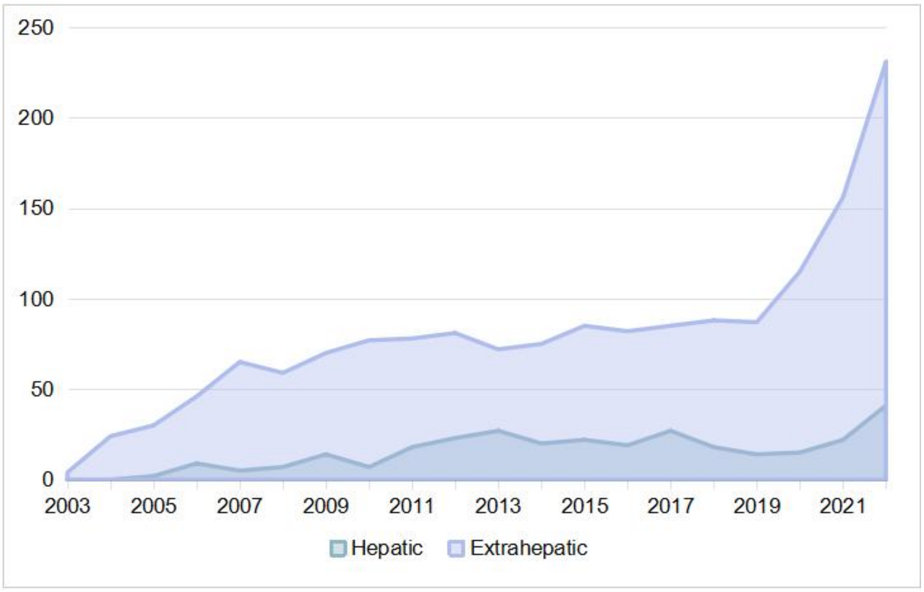

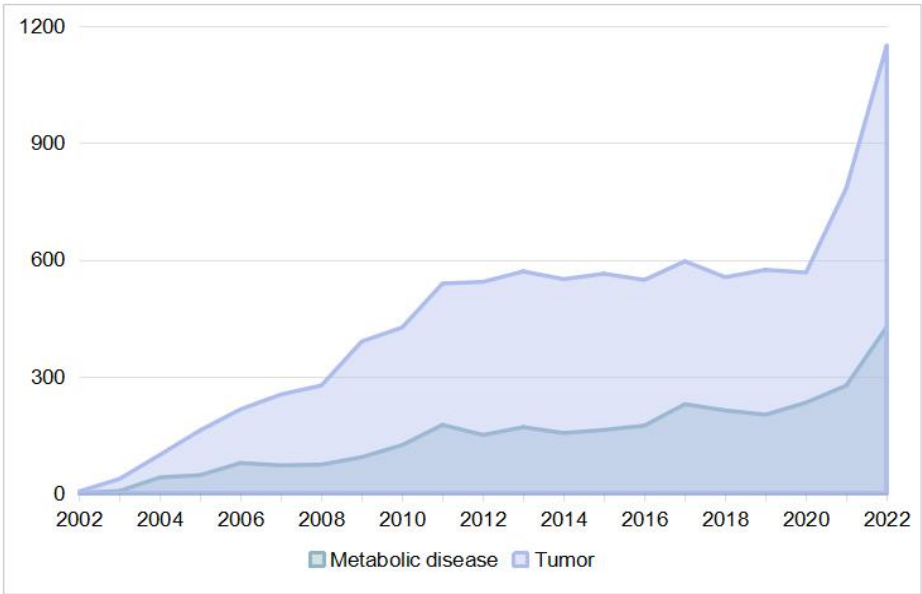

Figure S3. Time trajectories of delivery routes and disease categories in siRNA delivery patent

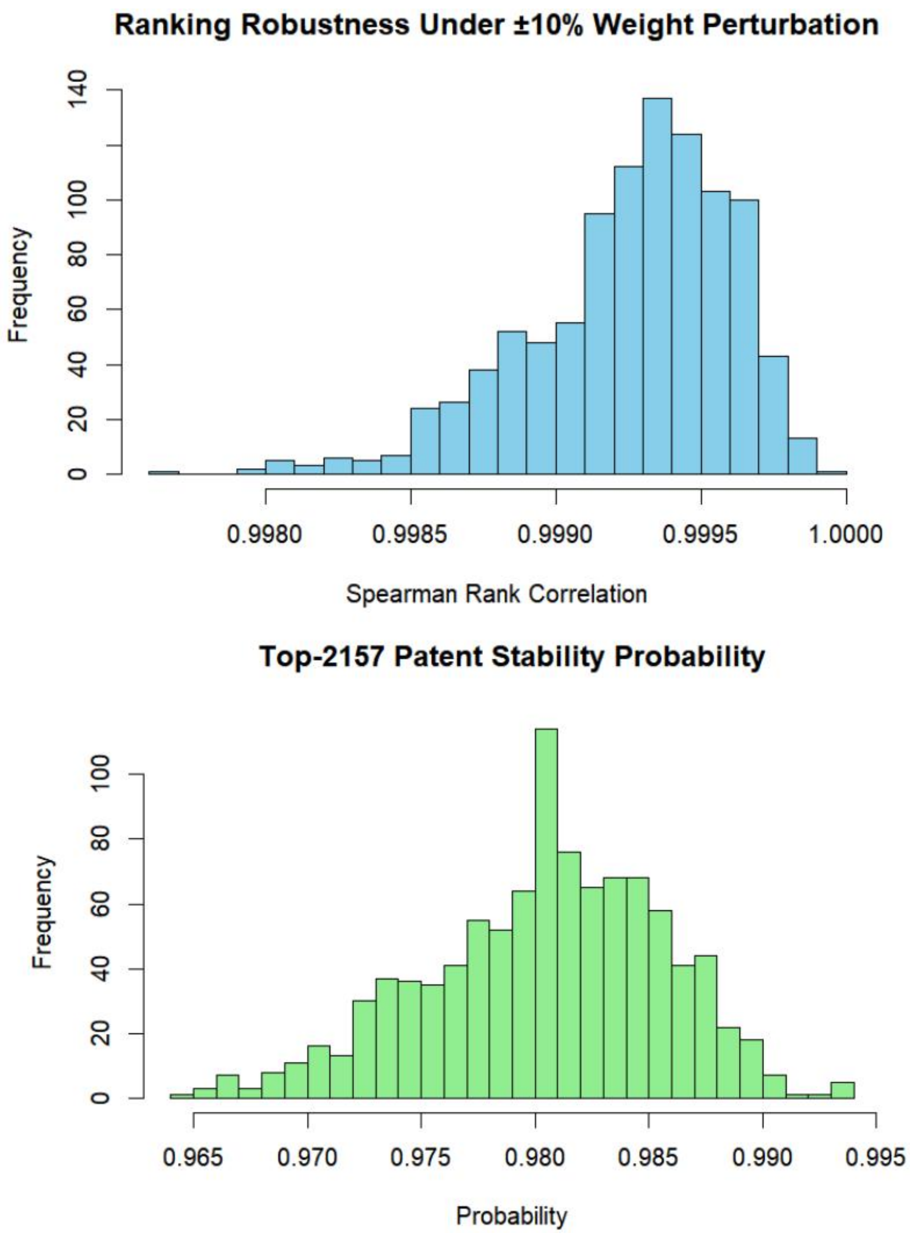

Figure S4. Stability of patent rankings under weight perturbations

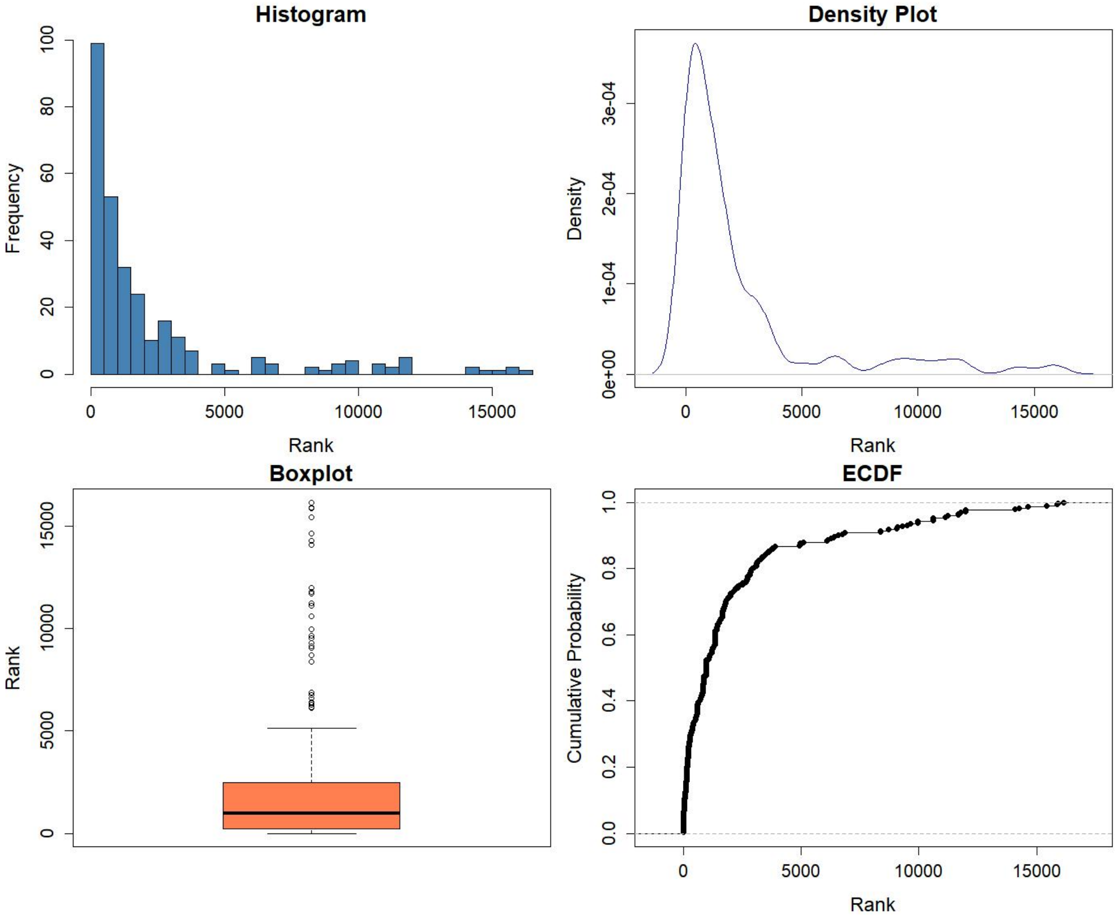

Figure S5. Distribution of validation patents in model score ranking

Supplemental Tables

Table S1. Quantification tables

<table>
<tr><td></td><td colspan="2">Technical perspective</td><td colspan="2">Legal perspective</td><td>Commerical perspective</td></tr>
<tr><td>Mean</td><td colspan="2">0.32</td><td colspan="2">0.27</td><td>0.41</td></tr>
<tr><td>Minimum</td><td colspan="2">0.11</td><td colspan="2">0.08</td><td>0.21</td></tr>
<tr><td>Maximun</td><td colspan="2">0.45</td><td colspan="2">0.48</td><td>0.58</td></tr>
<tr><td>SD</td><td colspan="2">0.08</td><td colspan="2">0.12</td><td>0.11</td></tr>
<tr><td>Disagreement</td><td colspan="5">0.091</td></tr>
<tr><td colspan="6">Legal perspective</td></tr>
<tr><td></td><td>Claims</td><td>Patent Family</td><td>Patent Age</td><td>Patent Geographic Coverage</td><td>Patent Scope</td></tr>
<tr><td>Mean</td><td>0.23</td><td>0.24</td><td>0.18</td><td>0.22</td><td>0.12</td></tr>
<tr><td>Minimum</td><td>0.11</td><td>0.19</td><td>0.12</td><td>0.14</td><td>0.06</td></tr>
<tr><td>Maximun</td><td>0.3</td><td>0.27</td><td>0.35</td><td>0.4</td><td>0.15</td></tr>
<tr><td>SD</td><td>0.06</td><td>0.02</td><td>0.06</td><td>0.08</td><td>0.03</td></tr>
<tr><td>Disagreement</td><td colspan="5">0.046</td></tr>
<tr><td colspan="6">Commercial perspective</td></tr>
</table>

	Primary Indication	Technological Accumulation	Organizational Category	Collaboration Status	R&D Experience
Mean	0.31	0.26	0.15	0.17	0.12
Minimum	0.09	0.15	0.04	0.06	0.02
Maximun	0.68	0.39	0.23	0.29	0.2
SD	0.17	0.07	0.06	0.07	0.05
Disagreement	0.086				

Technical perspective

	Technology Combination	Drug Delivery Method	Technological Impact	Translational Evidence	Field Position	Technology Maturity
Mean	0.12	0.25	0.19	0.19	0.16	0.09
Minimum	0.09	0.18	0.18	0.19	0.16	0.09
Maximun	0.16	0.28	0.2	0.2	0.17	0.1
SD	0.02	0.04	0.01	0	0	0
Disagreement	0.015					

Table S2. Distribution of component scores across clusters

Cluster	n	Overall	Technical	Commercial	Legal
Cluster 6	2157	72.064 (2.843)	18.709 (3.222)	32.568 (2.373)	20.788 (2.187)
Cluster 5	3772	64.957 (1.834)	15.813 (3.159)	30.012 (2.875)	19.132 (2.268)
Cluster 4	4346	59.181 (1.601)	14.170 (3.004)	27.284 (3.080)	17.727 (2.510)
Cluster 3	5086	53.906 (1.539)	13.066 (2.801)	24.636 (3.312)	16.205 (2.699)
Cluster 2	3377	48.245 (1.808)	12.227 (2.600)	21.600 (3.640)	14.418 (3.227)
Cluster 1	1580	41.016 (2.968)	11.534 (2.221)	17.483 (3.494)	12.000 (3.227)

Table S3. Component score distribution Detailed information on the top 50 ranked patent families (This table is provided separately as an Excel file due to its size. Please refer to the accompanying Excel file "Table_S3.xlsx" for the full dataset.)

Table S4. List of expert participants

Expert	Title	Background	Category
Expert 1	Program Manager	Industry	Middle manager
Expert 2	Program Manager	Industry	Middle manager
Expert 3	Program Manager	Industry	Senior manager
Expert 4	Program Manager	Industry	Senior manager
Expert 5	R&D Manager	Industry	Middle manager
Expert 6	R&D Manager	Industry	Senior manager
Expert 7	Intellectual Property Manager	Industry	Middle manager
Expert 8	Senior Consultant	Industry	Middle manager
Expert 9	Vice President of R&D	Industry	Senior manager
Expert 10	Vice President of R&D	Industry	Senior manager
Expert 11	CEO	Industry	Senior manager
Expert 12	CEO	Industry	Senior manager
Expert 13	Professor	Academia	Researcher
Expert 14	Professor	Academia	Researcher
Expert 15	Professor	Academia	Researcher
Expert 16	Postdoc	Academia	Assistant Researcher
Expert 17	Postdoc	Academia	Assistant Researcher
Expert 18	Postdoc	Academia	Assistant Researcher
Expert 19	Postdoc	Academia	Assistant Researcher
Expert 20	Medical Doctor	Academia	Assistant Researcher
Expert 21	Patent Examiner	Government	Junior Patent Examiner
Expert 22	Patent Examiner	Government	Senior Patent Examiner
Expert 23	Patent Examiner	Government	Senior Patent Examiner

Table S5. Chi-square test of patent classification and marketed product-related patents enrichment

Comparison	Group	Marketed patents	Non-marketed patents	P
1	Cluster 6	212	1945	1.9195E-239
	Cluster 2-5	79	16502	
2	Cluster 6	212	1945	1.10235E-37
	Cluster 1	0	1580	
3	Cluster 2-5	79	16502	0.005965208
	Cluster 1	0	1580	
