# Supplementary material for: Evaluation and forecasting of siRNA delivery technologies: An analysis of hierarchical decision model based on patent data
Source: Mol Ther Nucleic Acids. 2026 Apr 30;37(2):102943. doi: 10.1016/j.omtn.2026.102943 (PMC13213759; doi:10.1016/j.omtn.2026.102943)
Supplement: Data S2. Questionnaires [file mmc2.pdf]

Questionnaire 1

Dear Expert,

Hello!

Please open the following link to access a survey that requires your input:

http://research1.etm.pdx.edu/hdm2/Expert.aspx?ID=eab7a97ec81194d0/2d722f9d6ecaf9d0

You will be asked to compare the importance of different criteria by pairs and provide quantitative scores for each pair. For example:

- If criterion A is three times as important as criterion B, assign 75 points to A and 25 points to B.
- If A and B are equally important, assign 50 points to each.
- If A is one-fourth as important as B, assign 20 points to A and 80 points to B.
- Do not use 0 in pairwise comparisons. If A is negligible compared to B, assign 1 point to A and 99 points to B.

Please note:

1. **The survey consists of multiple sections. You will have completed the entire assessment only when the interface resembles Image Guide 7 shown below.**
2. **If you encounter any issues with moving the slider during scoring, please manually enter the score in the box provided.**

Below are the image guides and indicator tables for your reference. We greatly appreciate your professional advice.

Image Guide:

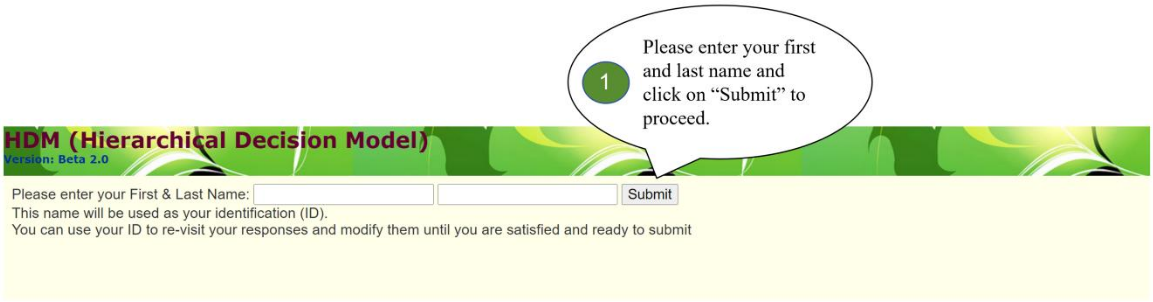
1
Please enter your first and last name and click on “Submit” to proceed.
HDM (Hierarchical Decision Model)
Version: Beta 2.0
Please enter your First & Last Name:
Submit
This name will be used as your identification (ID).
You can use your ID to re-visit your responses and modify them until you are satisfied and ready to submit

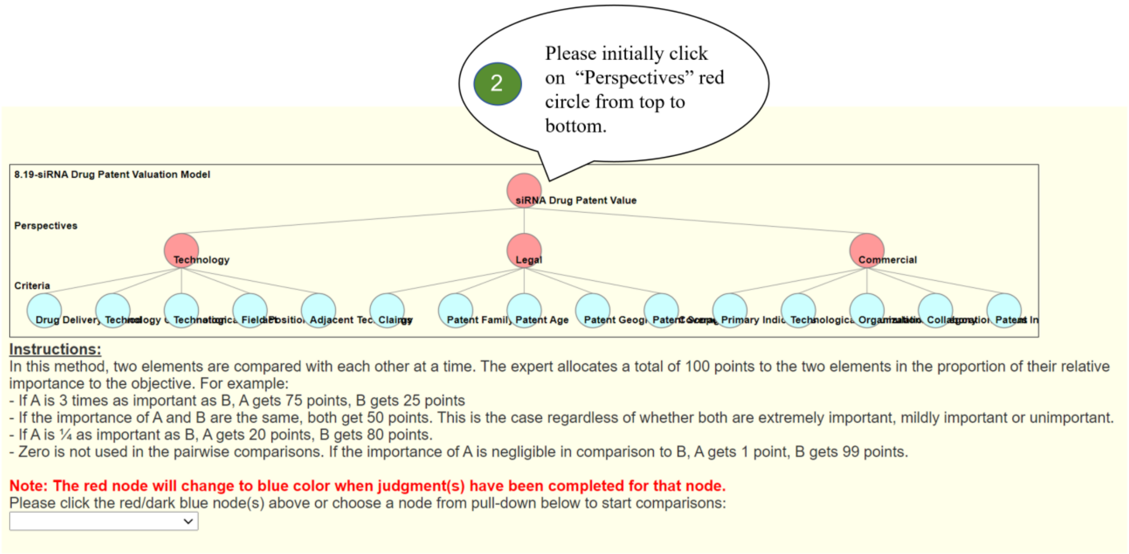
2
Please initially click on “Perspectives” red circle from top to bottom.
8.19-siRNA Drug Patent Valuation Model
siRNA Drug Patent Value
Perspectives
Technology
Legal
Commercial
Criteria
Patent Age
Instructions:
In this method, two elements are compared with each other at a time. The expert allocates a total of 100 points to the two elements in the proportion of their relative importance to the objective. For example:
- If A is 3 times as important as B, A gets 75 points, B gets 25 points
- If the importance of A and B are the same, both get 50 points. This is the case regardless of whether both are extremely important, mildly important or unimportant.
- If A is ¼ as important as B, A gets 20 points, B gets 80 points.
- Zero is not used in the pairwise comparisons. If the importance of A is negligible in comparison to B, A gets 1 point, B gets 99 points.
Note: The red node will change to blue color when judgment(s) have been completed for that node.
Please click the red/dark blue node(s) above or choose a node from pull-down below to start comparisons:

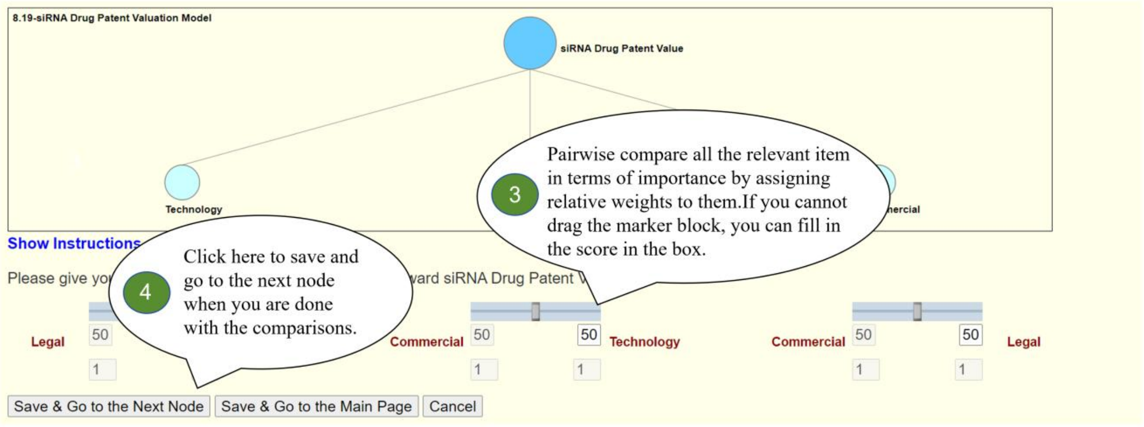
8.19-siRNA Drug Patent Valuation Model
siRNA Drug Patent Value
Technology
3
Pairwise compare all the relevant item in terms of importance by assigning relative weights to them.If you cannot drag the marker block, you can fill in the score in the box.
Show Instructions
4
Click here to save and go to the next node when you are done with the comparisons.
Legal
50
1
Commercial
50
50
Technology
1
1
Commercial
50
50
Legal
1
1
Save & Go to the Next Node
Save & Go to the Main Page
Cancel

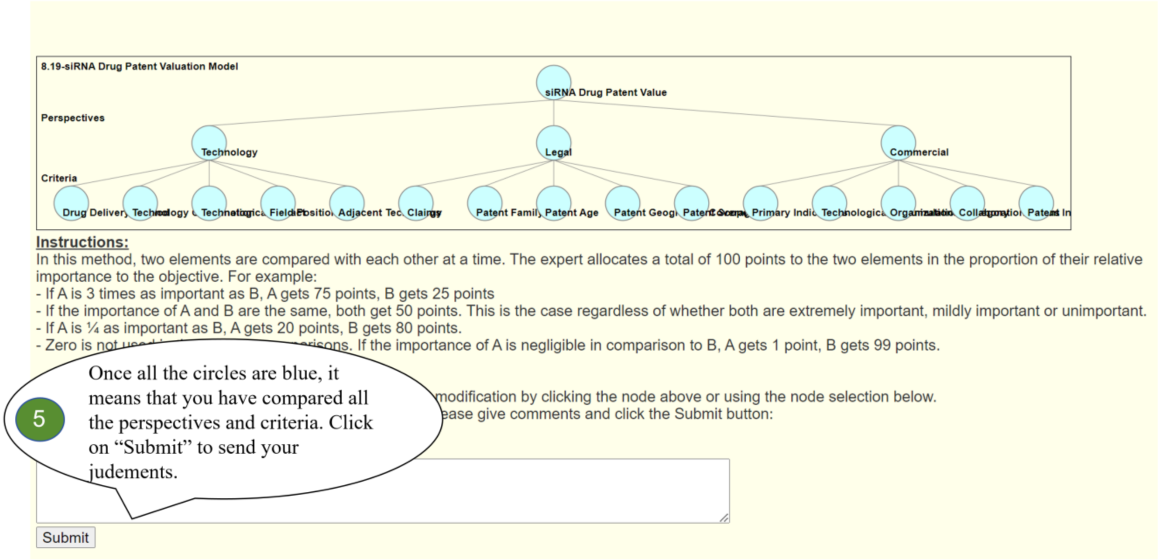
8.19-siRNA Drug Patent Valuation Model
siRNA Drug Patent Value
Perspectives
Technology
Legal
Commercial
Criteria
Patent Age
Instructions:
In this method, two elements are compared with each other at a time. The expert allocates a total of 100 points to the two elements in the proportion of their relative importance to the objective. For example:
- If A is 3 times as important as B, A gets 75 points, B gets 25 points
- If the importance of A and B are the same, both get 50 points. This is the case regardless of whether both are extremely important, mildly important or unimportant.
- If A is ¼ as important as B, A gets 20 points, B gets 80 points.
- Zero is not u... ...arisons. If the importance of A is negligible in comparison to B, A gets 1 point, B gets 99 points.
...modification by clicking the node above or using the node selection below.
...ease give comments and click the Submit button:
Submit
5
Once all the circles are blue, it means that you have compared all the perspectives and criteria. Click on "Submit" to send your judements.

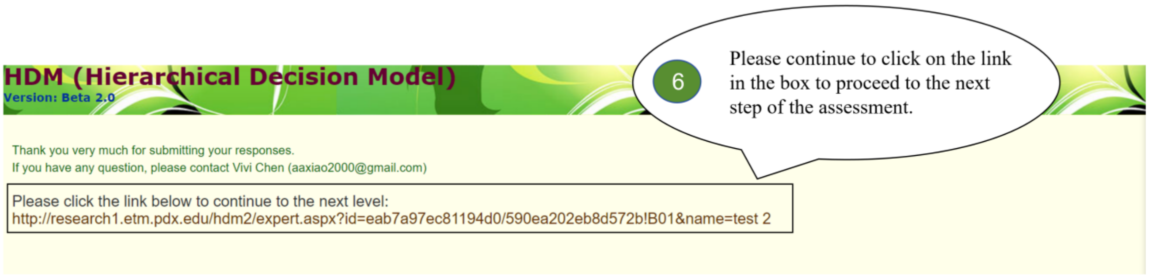
HDM (Hierarchical Decision Model)
Version: Beta 2.0
6
Please continue to click on the link in the box to proceed to the next step of the assessment.
Thank you very much for submitting your responses.
If you have any question, please contact Vivi Chen (aaxiao2000@gmail.com)
Please click the link below to continue to the next level:
http://research1.etm.pdx.edu/hdm2/expert.aspx?id=eab7a97ec81194d0/590ea202eb8d572b!B01&name=test 2

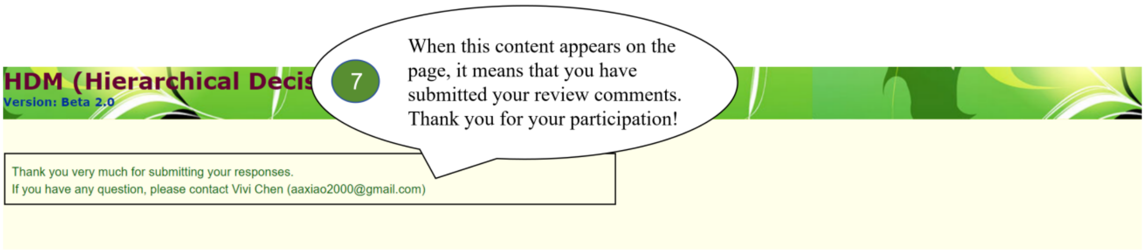
HDM (Hierarchical Decis
Version: Beta 2.0
7
When this content appears on the page, it means that you have submitted your review comments. Thank you for your participation!
Thank you very much for submitting your responses.
If you have any question, please contact Vivi Chen (aaxiao2000@gmail.com)

Questionnaire 2

Dear Expert,

Hello!

After successfully completing the first step of indicator weight allocation, we sincerely invite you to participate in the second phase of the evaluation. Please open the following link to access the survey interface, where your quantitative scoring is required:

Legal perspective:

https://umac.au1.qualtrics.com/jfe/form/SV_eX5BHoFhgqzFn7M

Technical and commercial perspectives:

https://umac.au1.qualtrics.com/jfe/form/SV_6VETQ9wq3xWbAJ8

Based on your professional experience, please assign a score between 0 and 100 for each indicator. Specifically:

0 points: Represents the lowest or least desirable level of the indicator. At this level, the indicator's performance is typically the poorest or contrary to the objective.

100 points: Represents the highest or most desirable level of the indicator. At this level, the indicator's performance is typically the best and most aligned with the objective.

After completing the evaluation, please review your responses carefully before submitting the final survey.

As you score the indicators, we strongly encourage you to reflect the relative importance of each indicator in the evaluation system by assigning the highest and lowest possible scores as appropriate.

Below is an image for your reference. We sincerely thank you again for your assistance!

Questionnaire screenshot:

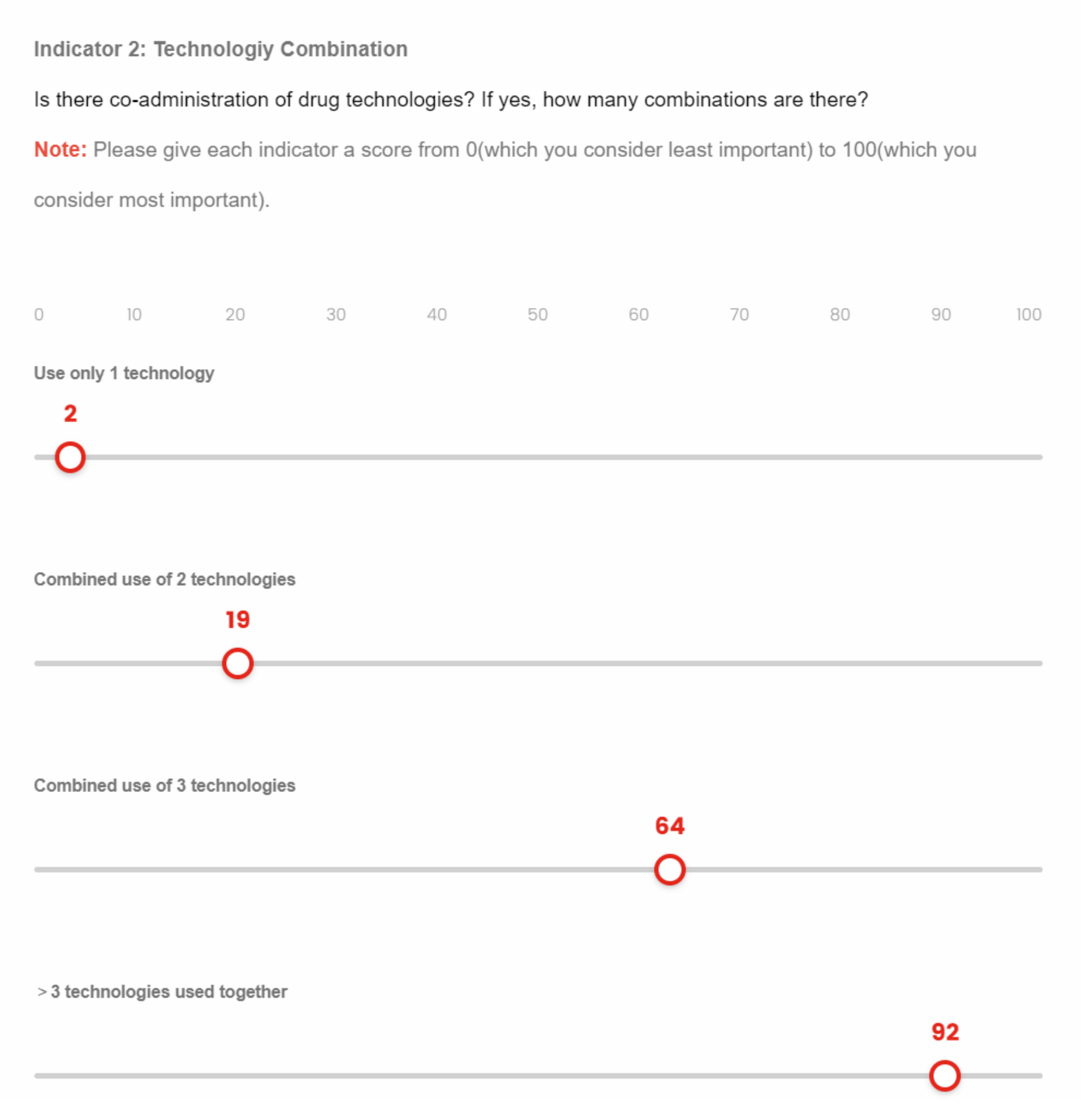
Indicator 2: Technologiy Combination

Is there co-administration of drug technologies? If yes, how many combinations are there?

Note: Please give each indicator a score from 0(which you consider least important) to 100(which you consider most important).

0 10 20 30 40 50 60 70 80 90 100

Use only 1 technology

2

Combined use of 2 technologies

19

Combined use of 3 technologies

64

> 3 technologies used together

92
